# Mapping of Human Autoantibody Binding Sites on the Calcium-Sensing Receptor

**DOI:** 10.1359/jbmr.090703

**Published:** 2009-07-06

**Authors:** E Helen Kemp, Nikos G Gavalas, Samia Akhtar, Kai JE Krohn, J Carl Pallais, Edward M Brown, Philip F Watson, Anthony P Weetman

**Affiliations:** 1 Department of Human Metabolism, School of Medicine, University of Sheffield Sheffield S10 2JF, United Kingdom; 2 Department of Pathology, Tampere University Hospital and Institute of Medical Technology, University of Tampere Tampere 33101, Finland; 3 Endocrine Unit, Massachusetts General Hospital Boston, MA, USA; 4 Division of Endocrinology, Diabetes and Hypertension, Department of Medicine, Brigham and Women's Hospital and Harvard Medical School Boston, MA, USA

**Keywords:** autoantibody, autoimmune polyendocrine syndrome type 1, parathyroid, calcium-sensing receptor, hypoparathyroidism

## Abstract

Previously, we have demonstrated the presence of anti-calcium-sensing receptor (CaSR) antibodies in patients with autoimmune polyglandular syndrome type 1 (APS1), a disease that is characterized in part by hypoparathyroidism involving hypocalcemia, hyperphosphatemia, and low serum levels of parathyroid hormone. The aim of this study was to define the binding domains on the CaSR of anti-CaSR antibodies found in APS1 patients and in one patient suspected of having autoimmune hypocalciuric hypercalcemia (AHH). A phage-display library of CaSR peptides was constructed and used in biopanning experiments with patient sera. Selectively enriched IgG-binding peptides were identified by DNA sequencing, and subsequently, immunoreactivity to these peptides was confirmed in ELISA. Anti-CaSR antibody binding sites were mapped to amino acid residues 41–69, 114–126, and 171–195 at the N-terminal of the extracellular domain of the receptor. The major autoepitope was localized in the 41–69 amino acid sequence of the CaSR with antibody reactivity demonstrated in 12 of 12 (100%) APS1 patients with anti-CaSR antibodies and in 1 AHH patient with anti-CaSR antibodies. Minor epitopes were located in the 114–126 and 171–195 amino acid domains, with antibody reactivity shown in 5 of 12 (42%) and 4 of 12 (33%) APS1 patients, respectively. The results indicate that epitopes for anti-CaSR antibodies in the AHH patient and in the APS1 patients who were studied are localized in the N-terminal of the extracellular domain of the receptor. The present work has demonstrated the successful use of phage-display technology in the discovery of CaSR-specific epitopes targeted by human anti-CaSR antibodies. © 2010 American Society for Bone and Mineral Research.

## Introduction

A complex homeostatic system involving the interplay of the bones, kidneys, and intestines has evolved to maintain extracellular concentrations of calcium within a relatively narrow range.(1) The primary regulator of this system is parathyroid hormone (PTH), the release of which is initiated by signals from the calcium-sensing receptor(2) (CaSR) in response to circulating calcium levels. When the CaSR is activated by elevated calcium, it signals the retention of PTH in the parathyroid cells. Conversely, when the CaSR is not activated owing to low calcium levels, it does not signal, and PTH is released. Over- and underproduction of PTH gives rise to hyper- and hypocalcemia, respectively, because the efflux of calcium from bone, the reabsorption of urinary calcium, and the uptake of dietary calcium are variously affected.(1)

Autoimmune polyendocrine syndrome type 1 (APS1) is a rare autosomal recessive disorder(3) caused by mutations in the autoimmune regulator (*AIRE*) gene(4,5) and is characterized by multiple organ-specific autoimmunity and ectodermal manifestations.(3,6) In the majority of cases, disease components include mucocutaneous candidiasis, hypoparathyroidism, and Addison's disease, with type 1 diabetes mellitus, alopecia, vitiligo, autoimmune hepatitis, and pernicious anemia occurring less frequently. Patients typically display a wide variety of autoantibodies against enzymes found in the affected organs, including 21-hydroxylase, 17-α-hydroxylase, and side-chain cleavage enzyme, all of which are present in the adrenal cortex,(7–9) as well as pancreatic glutamic acid decarboxylase 65 and tyrosine phosphatase-like protein IA-2, which are also prevalent in autoimmune type 1 diabetes mellitus.(10,11)

As part of APS1, hypoparathyroidism occurs in 80% of patients and is characterized by hypocalcemia, hyperphosphatemia, and low serum levels of PTH. Early reports suggested that the clinical symptoms and biochemical manifestations of hypoparathyroidism could result from humoral immune responses to parathyroid cells.(12,13) Subsequently, the G protein-coupled CaSR(2) was identified as a parathyroid autoantigen in patients with APS1,(14–16) as well as in individuals affected by isolated autoimmune hypoparathyroidism(14,15,17,18) and autoimmune hypocalciuric hypercalcemia (AHH).(19–21) Earlier studies have indicated that patient anti-CaSR antibodies recognize epitopes in the extracellular domain,(14–16) but only in a few cases has binding to specific CaSR peptides been demonstrated.(18–20)

The aim of this study was to define the binding sites of anti-CaSR antibodies detected in a cohort of patients with APS1 in a previous study.(16) In addition, the epitopes recognized by anti-CaSR antibodies in a single patient with putative AHH were to be determined. To achieve this objective, peptide phage-display technology was employed.

## Materials and Methods

### Patients and controls

The study was approved by the Ethics Committee of Tampere University Hospital, Tampere, Finland, and the APS1 patients participated in the study after obtaining informed consent, given either by the patient or by the patient's parents. The study of the patient with putative AHH was approved by the Institutional Review Board of Partners Health Care, Boston, MA, USA, and serum was obtained from the patient after obtaining written informed consent. Sera were stored at −20°C prior to use.

Fourteen APS1 patients (7 male, 7 female; mean age 18 years, with a range of 10 to 47 years) were studied. All patients had Addison's disease and mucocutaneous candidiasis. Thirteen patients had hypoparathyroidism. Other autoimmune diseases present were premature ovarian failure (5), alopecia (3), vitiligo (5), type 1 diabetes mellitus (3), autoimmune hypothyroidism (1), and pernicious anemia (2). All patients carried mutations in both alleles of the *AIRE* gene.(5) In our previous study, anti-CaSR antibodies were detected using immunoprecipitation assays in 12 of these patients.(16)

One AHH patient (female, age 73 years) with a positive antinuclear antibody titer of 1:5120 and anti-ribonuclear protein antibodies developed hypercalcemia, an elevated level of intact PTH and marked hypocalciuria (10 to 40 mg/24 h), prompting investigation of possible AHH. Three separate serum samples were available, and anti-CaSR antibodies were detected in each of them (unpublished data) using immunoprecipitation assays.(16)

Twenty healthy individuals (9 male, 11 female; mean age 32 years, with range 24 to 48 years) who had no present or past history of autoimmune disorders were included as controls. No individual had anti-CaSR antibodies when tested in immunoprecipitation assays.(16)

### Specific anti-CaSR antibodies

Anti-CaSR rabbit polyclonal antibody against a synthetic peptide corresponding to amino acids 12–27 of the rat CaSR was purchased from Alexis Biochemicals (Nottingham, UK). The antibody has cross-reactivity with the human CaSR. Anti-CaSR mouse monoclonal antibody against a synthetic peptide corresponding to amino acids 214–235 of the human CaSR was obtained from Acris Antibodies (Herford, Germany).

### Phage-display library construction

Vector pComb3(22) was used to construct a phage-display library of CaSR peptides. The vector is designed to allow the expression of cloned DNA fragments and the subsequent surface exposure of the peptides encoded therein on phage particles. For surface expression, DNA fragments are required to be cloned in frame with the PelB leader peptide and the gene III phage coat protein present in pComb3 at the N- and C-terminal, respectively.

To construct a CaSR cDNA fragment library in pComb3, full-length CaSR cDNA was prepared from pcCaSR-FLAG(16) by restriction of the plasmid with *Kpn*I (Promega) and *Xba*I (Promega). The resulting 3255-bp CaSR cDNA fragment was separated by agarose gel electrophoresis(23) and purified using a Wizard PCR Preps DNA Purification System (Promega, Southampton, UK). Random 100- to 300-bp fragments of CaSR cDNA were prepared by digestion with DNAse I in a reaction containing: 1 µg CaSR cDNA, 1 unit DNAse I (Promega), and DNAse I buffer (Promega). The reaction was incubated at room temperature for 15 minutes and then terminated by the addition of 50 mM EDTA (pH 8.0). The DNA fragments were purified using a Wizard PCR Preps DNA Purification System and treated with T_4_ DNA polymerase (Promega) according to the manufacturer's protocol to create blunt ends. After further purification, the fragments were ligated into the *Eco*RV restriction site of pComb3 using standard methods.(23) The CaSR cDNA fragment library was recovered by electroporation of *Escherichia coli* XL1-Blue cells (Stratagene, La Jolla, CA, USA), as described by the manufacturer. The library size was estimated by plating out samples of electroporated cells onto Luria-Bertani (LB) agar(23) containing 100 µg/mL ampicillin and 10 µg/mL tetracycline.

To prepare the CaSR peptide phage-display library, the electroporated cells were incubated for 1 hour at 37°C before superinfection with 1 × 10^12^ plaque-forming units of VCMS13 helper phage (Stratagene) at 37°C for 15 minutes. The culture subsequently was transferred to 100 mL of LB medium(23) supplemented with 100 µg/mL ampicillin, 10 µg/mL tetracycline, and 10 µg/mL kanamycin. After overnight incubation at 37°C, the culture was centrifuged and phage precipitated from the supernatant with 0.2 volumes of 20% (w/v) polyethylene glycol 4000/2.5 M NaCl. The phage were resuspended in 2 to 3 mL phosphate-buffered saline (pH 7.4; PBS, Sigma, Poole, UK) and stored at −20°C. The phage titer was determined by infecting log-phase *E. coli* XL1-Blue with an aliquot of the phage-display library and then plating out samples onto selective LB agar.

### Biopanning experiments

For biopanning experiments, human sera or animal anti-CaSR antibodies (10-µL aliquots) were applied to the wells of Corning polystyrene 96-well microtiter plates (Bibby Sterilin, Ltd., Mid Glamorgan, UK) in 50 µL of buffer containing 1.5 mM Na_2_CO_3_, 3.5 mM NaHCO_3_, and 3.0 mM NaN_3_ (pH 9.2). Plates were incubated at room temperature for 2 hours to allow antibody binding before washing with PBS/0.05% (w/v) Tween 20 (PBS/Tween). To block any nonspecific phage binding later in the procedure, 400 µL 2% (w/v) bovine serum albumin (BSA) in PBS was added to the wells, and incubation at room temperature continued for 2 hours. The wells were rinsed again with PBS/Tween before the addition of a 100-µL sample of phage-display library containing 1 × 10^10^ colony-forming units (cfus). Plates were incubated overnight at 4°C to allow the interaction of anti-CaSR antibodies with peptides displayed on the surfaces of the phage particles. The wells were washed extensively with PBS/Tween to remove unbound phage. Bound phage then were eluted with 150 µL 100 mM HCl (adjusted to pH 2.2 with solid glycine) and neutralized with 9 µL 2 M Tris-HCl (pH 7.6). The phage suspension was used subsequently to infect 2 mL of exponentially growing *E*. *coli* XL1-Blue cells for 15 minutes at room temperature. Aliquots of the infected cells then were plated onto selective medium to allow the recovery of individual bacterial clones for analysis.

To generate a phage-display library for a further round of selection, the infected *E*. *coli* XL1-Blue cell culture was superinfected with helper phage and phage precipitated and titred as described earlier. This first-round library enriched in phage displaying antibody-binding peptides then was used in a second round of selective enrichment. In all, five rounds of biopanning were undertaken.

For analysis, individual bacterial clones were cultured, and phagemid DNA was prepared using a Wizard Minipreps DNA Purification System (Promega). To confirm the presence of a cDNA insert, phagemid DNA (50-ng samples) was subjected to 36 cycles of polymerase chain reaction (PCR) amplification in a DNA Thermal Cycler with primers 5'-GGTGGCGGCCGCAAATTC-3' and 5'-GCCGCCAGCATTGACAGG-3' (MWG Biotech, Munich, Germany) using previously detailed reaction conditions.(24) The primers used flank the *Eco*RV cloning site in pComb3. The PCR amplification products were analyzed by agarose gel electrophoresis and purified according to a Wizard PCR Preps DNA Purification System (Promega). Sequencing with primer 5'-GGTGGCGGCCGCAAATTC-3' was carried using a BigDye Terminator Version 3.1 Cycle Sequencing Kit (Applied Biosystems, Foster City, CA, USA) and an ABI 3730 capillary sequencer (Applied Biosystems). Insert DNA sequences were compared with the full-length CaSR cDNA sequence using the BLAST network service of the National Center for Biotechnology Information (Bethesda, MD, USA).

### Phage ELISA

For increased expression of phage-displayed CaSR peptides for use in a phage ELISA, cDNA sequences encoding the CaSR peptides of interest were subcloned into vector pComb8(22) using restriction enzymes *Xho*I (Promega) and *Spe*I (Promega) employing standard subcloning protocols.(24) All constructs were verified by DNA sequencing, as detailed earlier.

Phage displaying CaSR peptides required for analysis in phage ELISA then were prepared from individual bacterial clones as described earlier after electroporating the appropriate phagemid DNA into *E. coli* XL1-Blue cells and superinfecting with helper phage. Corning polystyrene 96-well microtiter plates were coated with 100-µL aliquots of phage (10^10^ cfus) in coating buffer containing 1.5 mM Na_2_CO_3_ and 3.5 mM NaHCO_3_ (pH 9.2) and incubated overnight at 4°C. Helper phage were included in each assay as a control for background antibody binding. Wells were washed with PBS/0.1% (w/v) Tween 20, blocked with 3% (w/v) BSA in PBS at room temperature for 1 hour, and then washed with PBS/0.1% (w/v) Tween 20. Human sera were preabsorbed with *E. coli* extract and helper phage and then tested in phage ELISA at dilutions of 1:50. Animal anti-CaSR antibodies were analyzed at a dilution of 1:1000. Aliquots (100 µL) of serum/antibody were added to wells, and PBS was applied as a control. The plates were incubated at room temperature for 2 hours and then washed six times with PBS/0.1% (w/v) Tween 20. Aliquots (100 µL) of antihuman (Sigma), antirabbit (Sigma). or antimouse (Acris Antibodies) IgG alkaline phosphatase conjugate diluted 1:1000 in PBS/0.1% (w/v) Tween 20 were added to the wells for 1 hour at room temperature. After washing six times with PBS/0.1% (w/v) Tween 20, 100 µL of alkaline phosphatase substrate (Sigma Fast *p*-Nitrophenyl Phosphate Tablet Set, Sigma) were applied to each well, and plates were incubated at room temperature for 30 minutes. A LabSystems Integrated EIA Management System (Life Sciences International, Hampshire, UK) was used to read absorption of the wells at 405 nm.

All sera were tested in triplicate and the average OD_405_ value taken. OD_405_ values were corrected for background reactivity to helper phage to give an antibody (Ab) index. Each serum was tested in at least three experiments, and the mean Ab index was calculated. The upper limit of normal for each assay was calculated using the mean Ab index + 3 SD of the population of 20 healthy individuals. Any sample with an Ab index above the upper limit of normal was designated as positive for antibody reactivity to the phage-displayed CaSR peptide.

Synthetic peptides corresponding to amino acids 1–36, 41–69, 114–126, and 171–195 of CaSR were purchased from Severn Biotech, Ltd. (Kidderminster, UK). In absorption experiments, peptides were incubated with sera at 4°C for 2 hours at 1 mg/mL prior to testing the serum sample in phage ELISA. Ab indices then were calculated as before. The percentage antibody binding was determined as Ab index in presence of peptide/Ab index in absence of peptide × 100. Samples were tested in three experiments, and the mean percentage antibody binding was calculated.

### Synthetic peptide ELISA

Synthetic peptides corresponding to amino acids 214–238, 344–358, and 374–391 of CaSR were purchased from Severn Biotech, Ltd. Corning polystyrene 96-well microtiter plates were coated with 3 µg of peptide in coating buffer and incubated overnight at 4°C. Wells were washed with PBS/0.1% (w/v) Tween 20, blocked with 3% (w/v) BSA in PBS at room temperature for 1 hour, and then washed with PBS/0.1% (w/v) Tween 20. Human sera were tested in peptide ELISA at dilutions of 1:50. Aliquots (100 µL) of serum were added to wells, and PBS was applied as a control. The plates were incubated at room temperature for 2 hours and then washed six times with PBS/0.1% (w/v) Tween 20. Antibody binding was detected with alkaline phosphatase–conjugated secondary antibody, as detailed earliar.

All sera were tested in triplicate, and the average OD_405_ value was taken to give an Ab index. Each serum was tested in at least three experiments, and the mean Ab index was calculated. The upper limit of normal for each assay was calculated using the mean Ab index + 3 SD of the population of 10 healthy individuals. Any sample with an Ab index above the upper limit of normal was designated as positive for antibody reactivity to the CaSR peptide.

### Statistical analyses

The prevalence of antibody reactivity to phage-displayed and synthetic CaSR peptides was compared between patient groups and controls using Fisher's exact test for 2 × 2 contingency tables. Intra- and interassay variations were calculated as percentage coefficients of variation. Differences in antibody titers were analyzed by the Wilcoxon matched-pairs test. *P* values < .05 (two-tailed) were regarded as significant in all tests.

## Results

### Construction of a CaSR peptide phage-display library

A library of randomly generated CaSR cDNA fragments was constructed in the phage-display vector pComb3. The size of the CaSR cDNA fragment library was 1.2 × 10^6^ independent clones. Amplification by PCR of phagemid DNA isolated from individual bacterial clones demonstrated that the CaSR cDNA fragment library was 100% recombinant, with insert size ranging from less than 100 to 300 bp. The sequencing of 50 individual phagemid DNA indicated that the library contained random CaSR cDNA fragments with no bias for particular CaSR sequences.

Following amplification with helper phage, an initial phage-display library of 5 × 10^11^ cfus/mL was produced. In this library, peptides that exactly represent CaSR amino acid sequences can be displayed on the surfaces of phage particles, provided that their encoding cDNA sequence is cloned in frame with the PelB leader peptide and the gene III phage coat protein present in pComb3 at the N- and C-terminals, respectively.

### Biopanning of the phage-display CaSR peptide library

In order to enrich immunoreactive peptides recognized by anti-CaSR antibodies, the CaSR peptide phage-display library was immunoscreened in biopanning experiments with sera from 14 APS1 patients, 1 AHH patient, and 2 controls, as well as with anti-CaSR antibodies as positive controls. Five rounds of biopanning were carried out, and phagemid DNA from 16 to 20 bacterial clones was analyzed by DNA sequencing to identify CaSR peptides that had been selected during the enrichment process.

The results demonstrated that biopanning of sera from 12 of 14 (86%) APS1 patients and 1 AHH patient had enriched CaSR peptides containing a CaSR amino acid consensus sequence (Table 1). Nine of the 14 (64%) patients had enriched two consensus peptides, and 3 of 14 (21%) had enriched one consensus peptide (see Table 1). In all cases, the consensus peptide sequence was in frame with respect to both the PelB leader peptide and the gene III coat protein, indicating that the correct phage-surface expression of the encoded CaSR peptides could occur. In biopanning experiments with sera from 2 controls, no identifiable CaSR consensus sequence was enriched. This also was the case for 2 of 14 of the APS1 patient sera analyzed (see Table 1). In each case, phagemid from the fifth round of biopanning carried CaSR cDNA fragment inserts that would not be expressed correctly to give CaSR peptides on the surfaces of phage particles. The results also demonstrated that animal anti-CaSR polyclonal and monoclonal antibodies had enriched amino acid consensus sequences corresponding to the whole or part of the peptide against which they were raised (see Table 1).

**Table 1:** CaSR Consensus Peptide Sequences Enriched in Biopanning Experiments

Sample used in biopanning experiments^a^	CaSR consensus peptide sequences^b^	Amino acid residues of CaSR^c^	Number of clones with consensus sequence (%)
APS1-2	AQKKGDIILGGLFPIHFGVAAKDQDLKSRPESVECIRYNFRGFRWL	26–71	9/20 (45)
	TVSKALAEATLSFVAQNKIDSLNLDEFCNCSE	103–133	3/20 (15)
APS1-3	HFGVAAKDQDLKSRPESVECIRYNFRGFRWL	41–71	8/16 (50)
	KALAEATLSFVAQNKIDSLNLDEFCN	106–130	2/16 (13)
APS1-4	HFGVAAKDQDLKSRPESVECIRYNFRGFRWL	41-71	5/20 (25)
	FVAQNKIDSLNLD	114–126	3/20 (15)
APS1-5	LGGLFPIHFGVAAKDQDLKSRPESVECIRYNFRGFR	34–69	3/20 (15)
APS1-6	AQKKGDIILGGLFPIHFGVAAKDQDLKSRPESVECIRYNFRGFRWL	26-71	9/20 (45)
	SRLLSNKNQFKSFLRTIPNDEHQAT	171–195	2/20 (10)
APS1-7	GDIILGGLFPIHFGVAAKDQDLKSRPESVECIRYNFRGFR	30–69	8/20 (40)
	TVSKALAEATLSFVAQNKIDSLNLDEFCNCSE	103–133	3/20 (15)
APS1-8	No consensus peptide sequence defined	—	—
APS1-9	QKKGDIILGGLFPIHFGVAAKDQDLKSRPESVECIRYNFRGFRWL	27–71	2/20 (10)
APS1-10	AQKKGDIILGGLFPIHFGVAAKDQDLKSRPESVECIRYNFRGFRWL	26–71	10/20 (50)
	SRLLSNKNQFKSFLRTIPNDEHQAT	171–195	3/20 (15)
APS1-11	QKKGDIILGGLFPIHFGVAAKDQDLKSRPESVECIRYNFRGFRWL	27–71	2/20 (10)
APS1-12	No consensus peptide sequence defined	—	—
APS1-13	HFGVAAKDQDLKSRPESVECIRYNFRGFRWL	41–71	10/20 (50)
	SRLLSNKNQFKSFLRTIPNDEHQAT	171–195	3/20 (15)
APS1-14	HFGVAAKDQDLKSRPESVECIRYNFRGFRWL	41–71	8/20 (40)
	SRLLSNKNQFKSFLRTIPNDEHQAT	171–195	2/20 (10)
APS1-15	QKKGDIILGGLFPIHFGVAAKDQDLKSRPESVECIRYNFRGFRWL	27–71	3/20 (15)
	TVSKALAEATLSFVAQNKIDSLNLDEFCNCSE	103–133	3/20 (15)
AHH	HFGVAAKDQDLKSRPESVECIRYNFRGFRWL	41–71	9/20 (45)
Anti-CaSR Ab^d^	YGPDQRAQ	20–27	10/20 (50)
Anti-CaSR mAb^e^	ADDDYGRPGIEKFREEAEERDICI	214–237	10/20 (50)

a APS1 samples 2 to 15 are sera from APS1 patients; AHH sample is a serum sample from the single AHH patient.

b Peptide sequences enriched by biopanning of the phage-display CaSR peptide library with APS1 and AHH patient sera and animal anti-CaSR antibodies.

c Amino acid residues are numbered according to the CaSR peptide sequence with the ATG initiation codon as residue number 1.

d Polyclonal CaSR antibody.

e Monoclonal CaSR antibody.

Comparison of the consensus peptides enriched by each of the APS1 patient sera and the single AHH patient serum enabled identification of consensus sequences enriched by different patients (Table 2). Three consensus peptides were determined, CaSR amino acid residues 41–69, 114–126, and 171–195, these potentially representing separate antibody-binding sites on the receptor.

**Table 2:** CaSR Consensus Peptide Sequences Enriched by Different APS1 Patient Sera

Consensus peptide (amino acid residues of CaSR^a^)	Number of APS1 patient sera enriching consensus peptide (%)	APS1 patient serum samples enriching consensus peptide^c^
HFGVAAKDQDLKSRPESVECIRYNFRGFR (41–69)^b^	12/14 (86)	2–7, 9–11, 13–15
FVAQNKIDSLNLD (114–126)	5/14 (36)	2–4, 7, 15
SRLLSNKNQFKSFLRTIPNDEHQAT (171–195)	4/14 (29)	6, 10, 13, 14

a Amino acid residues are numbered according to CaSR peptide sequence with the ATG initiation codon as residue number 1.

b The 41–69 peptide sequence was enriched by a serum sample from the single AHH patient.

c Numbers refer to individual APS1 patients.

### Phage ELISA with patient and control sera

To confirm immunoreactivity to phage-displayed CaSR peptides identified in biopanning experiments, sera from 14 APS1 patients, 1 AHH patient (3 separate serum samples), and 20 healthy controls were tested in a phage ELISA format. All CaSR peptides used in phage ELISA were expressed in pComb8 following subcloning of the relevant cDNA fragment in frame with the PelB leader peptide and the gene VIII coat protein present in the vector. The phage used expressed either CaSR peptides 41–74, 110–130, or 169–198 because these encompassed the putative CaSR epitope consensus sequences 41–69, 114–126, and 171–195, respectively (Table 2). All sera also were analyzed in phage ELISA against peptides 1–36 and 210–241 that contained the epitopes for anti-CaSR polyclonal and monoclonal antibodies, respectively. Anti-CaSR antibody and anti-CaSR mAb were used as positive controls in phage ELISA against phage displaying either peptide 1–36 or peptide 210–241, respectively. An antibody index was calculated for each serum, and the upper limit of normal for each phage ELISA was determined. Any serum sample with an antibody index above the upper limit of normal was designated as positive for antibody reactivity to the phage-displayed CaSR peptide.

**Fig. 1:**
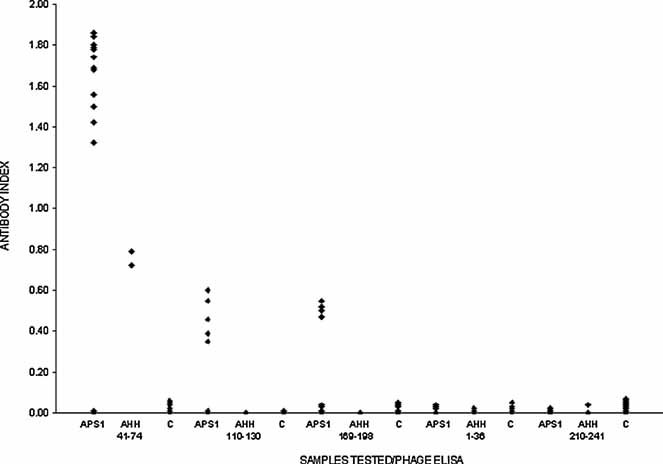
Phage ELISAs with patient and control sera. Patient and control sera were analyzed for antibody binding in phage ELISAs, as detailed in Materials and Methods. The Ab indices are shown for APS1 (*n* = 14) and AHH (*n* = 3) patient sera and for control (C) (*n* = 20) serum samples analyzed in phage ELISAs against phage-displaying CaSR peptides 1–36, 41–74, 110–130, 169–198, and 210–241.

The antibody indices for APS1 and AHH (3 separate serum samples) patients as well as control sera are shown in Fig. 1. Intra- and interassay variations for each sample were no more than 12% and 15%, respectively. All control sera were negative for antibody reactivity against all the phage-displayed CaSR peptides tested. The anti-CaSR polyclonal and monoclonal antibodies were positive only in phage ELISA against phage-displayed peptides 1–36 and 210–241, respectively.

Of the APS1 patient sera analyzed, 12 of 14 (86%) were positive for antibodies against at least one CaSR peptide (Table 3). Nine of the 14 (64%) patients were positive for antibodies against two CaSR peptides, and 3 of 14 (21%) were positive against one (see Table 3). None of the APS1 sera analyzed was positive for antibodies against peptides 1–36 and 210–241 (see Table 3). The two APS1 patient serum samples that failed to react against any phage-displayed CaSR peptides in phage ELISA were those that also failed to enrich specific sequences in biopanning experiments. Antibodies against CaSR peptides 41–74, 110–130, and 169–198 were significantly more prevalent in the APS1 patient group compared with controls (*P* < .0001, *P* = .0072 and *P* = .0216, respectively; see Table 3). The three separate serum samples from the AHH patient reacted only against CaSR peptide 41–74.

**Table 3:** Results of Phage ELISA

Peptide sequence displayed on phage^a^	Number of APS1 patient sera positive for antibody in phage ELISA (%)	Number of control sera positive for antibody reactivity in phage ELISA (%)	P value^b^	APS1 patient samples with antibody reactivity to peptide^c^
41–74	12/14 (86)	0/20 (0)	<0.0001	2–7, 9–11, 13–15
110–130	5/14 (36)	0/20 (0)	0.0072	2–4, 7, 15
169–198	4/14 (29)	0/20 (0)	0.0216	6, 10, 13, 14
1–36	0/14 (0)	0/20 (0)	—	—
210–241	0/14 (0)	0/20 (0)	—	—

a Amino acid residues are numbered according to CaSR peptide sequence with the ATG initiation codon as residue number 1.

b The prevalence of antibody reactivity to phage-displayed CaSR peptides was compared between APS1 patients and controls using Fisher's exact test for 2 × 2 contingency tables. *P* values < .05 (two-tailed) were regarded as significant.

c Numbers refer to individual APS1 patients.

To confirm antibody reactivity detected in phage ELISAs, APS1 patient sera, as appropriate, were incubated with and without synthetic peptides (41–69, 114–126, and 171–195) prior to testing in phage ELISA experiments. The CaSR peptide 1–36 was used as a control in all experiments. Antibody indices and the mean percentage antibody binding were calculated for each sample.

Antibody binding in phage ELISAs 41 to 74 in the presence of peptide 41–69 was reduced to 30% ± 4% for sera from APS1 patients 2 to 7, 9 to 11, and 13 to 15, as well as 26% ± 5% for the three serum samples from the AHH patient. In phage ELISAs 110 to 130 in the presence of peptide 114–126, antibody binding was reduced to 28% ± 7% for sera from APS1 patients 2, 3, 4, 7, and 15. In phage ELISAs 169 to 198 in the presence of peptide 171–195, antibody binding was reduced to 30% ± 7% for sera from APS1 patients 6, 10, 13, and 14. No inhibition of antibody binding was apparent in the presence of peptide 1–36 in any of the ELISAs.

### Analysis of CaSR epitopes for patient anti-CaSR antibodies

Analysis of the results from phage ELISAs suggested that a major CaSR epitope was localized in the N-terminal extracellular domain of the CaSR at amino acid residues 41–69 with antibody reactivity demonstrated in 12 of 12 (100%) of APS1 patients and the single patient with AHH (Table 4). Minor epitopes were located in amino acid domains 114–126 and 171–195, with antibody reactivity shown in 5 of 12 (42%) and 4 of 12 (33%) APS1 patients, respectively (see Table 4). Epitopes in amino acid sequences representing the remainder of the extracellular region, the membrane-spanning segment, and the intracellular domain were not identified. Multiple binding sites on CaSR were apparent for antibodies in 9 of 12 (75%) patients, and one binding site was identified in 3 of 12 (25%) patients.

**Table 4:** Epitope Specificities of APS1 Patient Anti-CaSR Antibodies

CaSR epitope^a^	Number of APS1 patients with anti-CaSR antibodies recognizing epitope (%)	APS1 patients with anti-CaSR antibodies recognizing epitope^c^
41–69^b^	12/12 (100)	2–7, 9–11, 13–15
114–126	5/12 (41)	2–4, 7, 15
171–195	4/12 (33)	6, 10, 13, 14

a Amino acid residues are numbered according to CaSR peptide sequence with the ATG initiation codon as residue number 1.

b The 41–69 epitope was recognized by anti-CaSR antibodies in the single AHH patient studied.

c Numbers refer to individual APS1 patients.

### Synthetic peptide ELISAs with patient and control sera

**Fig. 2:**
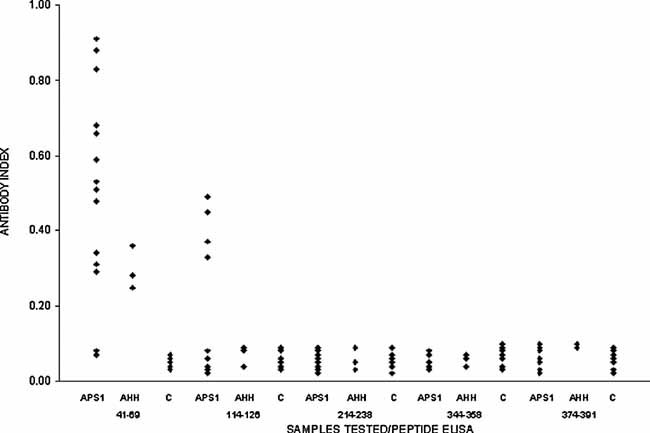
Synthetic peptide ELISAs with patient and control sera. Patient and control sera were analyzed for antibody binding to synthetic peptides in an ELISA format, as detailed in Materials and Methods. The Ab indices are shown for APS1 (*n* = 14) and AHH (*n* = 3) patient sera and for control (*C*) (*n* = 10) serum samples analyzed in ELISA experiments against synthetic CaSR peptides 41–69, 114–126, 214–238, 344–358, and 374–391.

To analyze immunoreactivity to CaSR synthetic peptides 41–69, 114–126, 214–238, 344–358, and 374–391, sera from 14 APS1 patients, 1 AHH patient (3 separate serum samples), and 10 healthy controls were tested in an ELISA format. An antibody index was calculated for each serum, and the upper limit of normal for each peptide ELISA was determined. Any serum sample with an antibody index above the upper limit of normal was designated as positive for antibody reactivity to the CaSR peptide. The anitbody indices for APS1 and AHH (3 separate serum samples) patients as well as control sera are shown in Fig. 2. Intra- and interassay variations for each sample were no more than 10% and 12%, respectively.

All control sera were negative for antibody reactivity against all the CaSR peptides tested. The three separate serum samples from the AHH patient reacted only against CaSR peptide 41–69. Of the APS1 patient sera analyzed, all were negative for antibody reactivity to peptides 214–238, 344–358, and 374–391. Antibody reactivity was evident against peptides 41–69 and 114–126 in 12 (patients 2 to 7, 9 to 11, and 13 to 15) and 5 (patients 2 to 4, 7, and 15), respectively, of the APS1 patient sera tested. The immunoreactivity of the APS1 patient sera in the peptide ELISAa confirmed that demonstrated in the phage ELISA experiments.

## Discussion

Previously, we identified anti-CaSR antibodies in patients with APS1 using immunoprecipitation assays.(16) The aim of this study was to define the epitope specificity of patient anti-CaSR antibodies using peptide phage display, a method that has been employed successfully in identifying autoantigenic epitopes in several autoimmune diseases.(25–27)

In the present study, anti-CaSR antibody-binding sites were mapped to amino acid residues 41–69, 114–126, and 171–195 at the N-terminal of the extracellular domain of the receptor, these being previously unreported. The major autoepitope appeared to be localized in amino acid sequence 41–69 of the CaSR, with antibody reactivity demonstrated in 12 of 12 (100%) of APS1 patients and the single AHH patient available for study. Minor epitopes were located in amino acid domains 114–126 and 171–195, with antibody reactivity shown in 5 of 12 (42%) and 4 of 12 (33%) of APS1 patients, respectively. Epitopes in the amino acid sequences representing the remainder of the extracellular region, the membrane spanning segment, and the intracellular domain of the CaSR were not identified. The results also indicate that the humoral response to the CaSR in APS1 is heterogeneous in nature, with several patients exhibiting antibodies to more than one CaSR epitope.

Overall, our findings are in agreement with earlier studies that have suggested that antigenic epitopes for human anti-CaSR antibodies are localized to the extracellular domain of the receptor.(14–16) However, specific epitopes at amino acids 214–236, 374–391, and 344–358 in the extracellular domain, which had been reported previously for patient antibodies targeting the CaSR,(18–20) were not identified in the present study. Similar results to ours have been reported recently for an AHH patient with anti-CaSR antibodies: No antibody reactivity to CaSR peptides 214–236, 374–391, and 344–358 was apparent in this individual.(21) In this study, the CaSR epitopes mapped using phage display were confirmed in ELISA experiments using synthetic peptides, indicating that both techniques can identify relevant antibody-binding sites. Variations between the epitopes identified in this study and those reported previously more likely may reflect differences between the individual patients who were included.

Interestingly, the major epitope (amino acids 41–69) demonstrated in this study overlaps the CaSR loop 1 domain (amino acids 50–59), which, if deleted, reduces receptor activation.(28,29) Binding of antibody to this epitope, therefore, might modulate the function of the receptor. Our data do not explain why the presence of autoantibodies to this major epitope should be associated with hypocalcemia in APS1 patients and hypercalcemia in the case of AHH. Perhaps binding of the antibody to separate regions within this epitope can exert diametrically opposite effects on the activity of the CaSR. It will be of considerable interest to study additional patients with AHH. However, such patients are extremely rare, and the patient studied here represents only the seventh identified to date with this condition.(19–21)

The minor epitope (amino acids 114–126) overlaps the CaSR loop 2 domain (amino acids 117–136). Deletion of this region or point mutations present in this region in the human disease autosomal dominant hypoparathyroidism (ADH) increase the sensitivity of the CaSR to Ca^2+^.(28,29) It has been postulated, therefore, that loop 2 may play a key role in maintaining the CaSR in an inactive state in part through the two disulfide linkages involving cysteine residues C129 and C131. Notably, mutations of these two amino acids are a cause of ADH in at least five families (see Calcium-Sensing Receptor Database at http://www.casrdb.mcgill.ca). In view of the foregoing, antibody binding to epitopes within loop 2 could either activate or inhibit the receptor depending on whether binding of the antibody favored the active or inactive conformation(s), respectively.

The minor epitope encompassing amino acid residues 171–195 is of particular interest because molecular modeling combined with site-directed mutagenesis has suggested that several of these residues are part of a binding site for extracellular calcium that lies in the crevice between the two lobes of each receptor monomer.(30,31) The residues that have been predicted to participate in calcium binding are amino acids S147, S170, D190, Y218, and E297. In addition, residues 169–171 are thought to be part of a binding site for amino acids that serve as allosteric activators of the receptor,(32) particularly phenylalanine, tyrosine, and other aromatics. It is easy to conceive of how binding of an antibody to this region of the receptor could perturb the interaction of calcium ions with this binding site and associated changes in the conformation of the CaSR's extracellular domain.

Since patient sera were used in this study to identify CaSR epitopes, it is not possible to discriminate between a single anti-CaSR antibody targeted at an epitope and a set of closely related anti-CaSR antibodies directed at the same epitope. To gain information on the epitope specificity of a particular autoantibody usually requires the production of human monoclonal antibodies from the patient. Indeed, monoclonal antibodies isolated from individuals with type 1 diabetes mellitus have been employed successfully in identifying the antibody-binding sites on glutamic acid decarboxylase.(33) In future work, the isolation of anti-CaSR monoclonal antibodies from APS1 patients will allow a more complete and detailed analysis of the array of anti-CaSR antibodies and the epitopes they recognize.

To summarise, the present study is the first to define specific epitopes on the CaSR for anti-CaSR antibodies in patients with the APS1, as well as identifying antibody-binding sites on the receptor in a case of AHH. In addition, we have demonstrated the successful use of phage-display technology in the discovery of CaSR epitopes for human antibodies that target this molecule. This may be of interest to other researchers who wish to define the binding sites for human anti-CaSR antibodies found in other diseases as well as monoclonal antibodies raised against the receptor.(34)
